# Characterisation of peripheral and central components of the rat monoiodoacetate model of Osteoarthritis

**DOI:** 10.1016/j.joca.2018.12.017

**Published:** 2019-04

**Authors:** S.M. Lockwood, D.M. Lopes, S.B. McMahon, A.H. Dickenson

**Affiliations:** † Department of Neuroscience, Physiology and Pharmacology, University College London, London, United Kingdom; ‡ Wolfson CARD, Hodgkin Building, Kings College London, London, United Kingdom

**Keywords:** MIA model, Diffuse noxious inhibitory controls, Descending controls

## Abstract

**Objective:**

Pain is the main reason patients report Osteoarthritis (OA), yet current analgesics remain relatively ineffective. This study investigated both peripheral and central mechanisms that lead to the development of OA associated chronic pain.

**Design:**

The monoiodoacetate (MIA) model of OA was investigated at early (2–6 days post injection) and late (>14 days post injection) time points. Pain-like behaviour and knee histology were assessed to understand the extent of pain due to cartilage degradation. Electrophysiological single-unit recordings were taken from spinal wide dynamic range (WDR) neurons to investigate Diffuse Noxious Inhibitory Controls (DNIC) as a marker of potential changes in descending controls. Immunohistochemistry was performed on dorsal root ganglion (DRG) neurons to assess any MIA induced neuronal damage. Furthermore, qPCR was used to measure levels of glia cells and cytokines in the dorsal horn.

**Results:**

Both MIA groups develop pain-like behaviour but only late phase (LP) animals display extensive cartilage degradation. Early phase animals have a normally functioning DNIC system but there is a loss of DNIC in LP animals. We found no evidence for neuronal damage caused by MIA in either group, yet an increase in IL-1β mRNA in the dorsal horn of LP animals.

**Conclusion:**

The loss of DNIC in LP MIA animals suggests an imbalance in inhibitory and facilitatory descending controls, and a rise in the mRNA expression of IL-1β mRNA suggest the development of central sensitisation. Therefore, the pain associated with OA in LP animals may not be attributed to purely peripheral mechanisms.

## Introduction

Pain is the major symptom of OA and the reason patients report the disease,^1^ yet the discordance between radiographic joint damage observed and the associated pain renders it difficult to understand which aspects of the disease trigger pain.^2^ Animal models of OA provide powerful tools to investigate mechanisms how structural joint pathology progresses and the associated development of chronic pain.^3^ An OA-like phenotype can be induced in animals by chemically injuring the joint with monoiodoacetate (MIA), causing chondrocyte cell death and articular cartilage erosion.^4^, ^5^, ^6^ The MIA model is particularly relevant for the pain associated with OA as animals develop hyperalgesia and allodynia in primary and secondary sites.^7^, ^8^, ^9^ Furthermore, the MIA model has many advantages including the lack of invasiveness, the simplicity of induction, the pain-like behaviour that develops, and the histological changes that occur in the articular cartilage.^10^

Diffuse noxious inhibitory controls (DNIC) are a unique form of endogenous inhibitory control of spinal wide dynamic range (WDR) neurons, where both noxious and innocuous evoked neuronal activity can be strongly inhibited by a second concomitant noxious conditioning stimulus outside of the receptive field.^11^, ^12^, ^13^ The activation of supraspinal structures and functional descending controls are required for DNIC to induce neuronal inhibition, as DNIC cannot be observed in anesthetized animals with spinal cord (SC) transection.^14^, ^15^ Specifically, the conditioning noxious stimulus activates descending inhibitory noradrenergic transmission to the SC, and neuronal inhibition is mediated by a final post-synaptic mechanism.^13^, ^16^ DNIC is lost in a neuropathic model, confirming that an imbalance in descending inhibitory and facilitatory controls are contributing to the maintenance of chronic pain.^13^, ^17^, ^18^, ^19^ Similarly, the human counterpart of DNIC, Conditioned Pain Modulation (CPM), has been demonstrated to be lost in a variety of chronic pain states.^20^, ^21^ Furthermore, measuring CPM responses in patients has been shown to predict the likelihood of patients developing chronic pain and how responsive they will be to drugs that restore this descending inhibition.^22^, ^23^ Overall, assessing DNIC or CPM provides a useful method for investigating the functionality of descending controls.

Variations with monoaminergic descending pathways have been demonstrated over the course of the MIA model, so this study investigated DNIC in both the early inflammatory phase (EP) and late chronic phases (LP) of the model.^24^ Furthermore, a neuropathic component of the MIA model has previously been reported, so MIA induced neuropathy was explored further to investigate if this was contributing to dysregulation of descending controls.^25^

## Methods

### Animals

In all experiments, male Sprague Dawley rats were used. Food and water were provided ab libitum, with cages kept in a 12 h light/dark cycle. All experiments were performed in accordance with the UK Animals (Scientific Procedures) Act 1986.

### The MIA model

Male Sprague–Dawley rats (190–210 g for early phase (EP) and 120–140 g for late phase) were anaesthetized with isofluorane, the ventral surface of the hind limb was clipped of hair and the knee area cleaned with clorhexidine. Arthritis was induced by an intrarticular injection of 2 mg MIA (Sigma) in 25 μL of 0.9% saline, with a 27 g needle into the left knee. Sham animals were assigned randomly and received an intrarticular injection of 25 μL 0.9% saline only, and assessed at the same time points as MIA animals.

### Behaviour

For all behavioural testing the experimenter was blinded to experimental or control groups. Testing was carried out on days 2, 4, 7 and 14 post injection. Animals were acclimatized to the equipment before testing. Mechanical hypersensitivity was tested using the up-down method as previously described (EP n = 10, EP sham (EPS) n = 10, late phase (LP) n = 10, LP sham (LPS) n = 10).^26^, ^27^ Von Frey filaments were applied to the plantar hind paw and withdrawal flinching or shaking was counted as a positive response. Weight bearing assessment was carried out using an incapacitance tester (Linton Instrumentation) (EP n = 15, EPS n = 12, LP n = 14, LPS n = 14). With one hind paw in contact with each force platform, the force exerted by each hind paw was measured over a 5 s period and repeated 3 times per animal. The average weight placed on the ipsilateral limb was quantified as a percentage of both hind paws.

### Electrophysiology

Electrophysiological experiments were carried out 2–6 days post MIA injection for EP animals (n = 18) and 14–20 days post injection for LP animals (n = 35) as previously described (Urch and Dickenson 2003). Briefly, animals were anesthetized for the duration of the experiment with isofluorane (1.5%) delivered in a gaseous mix of O_2_ (33%) and N_2_O (66%). A laminectomy was performed to expose the L4-L5 segments of the SC. Extracellular single unit recordings were made from deep dorsal horn WDR neurons (Lamina V-VI) using parylene coated tungsten electrodes (A-M systems). All WDR neurons used in this study responded to both innocuous and noxious stimulations in a graded manner coding intensity. Data was captured and analysed by a CED 1401 interface coupled to a computer running Spike2 software (Cambridge Electronic Design; rate functions).

### DNIC study design

Firstly, the pre-conditioned mechanically evoked neuronal firing rates were quantified in response to 8 g, 26 g and 60 g von Frey filament stimulation applied to the hind paw. This was repeated 3 times to obtain a stable pre-conditioned response (where all neurons met the inclusion criteria of <10% variation in action potential firing). For the DNIC response, the von Frey filaments were applied to the receptive field with a concurrent noxious ipsilateral ear pinch (15.75×2.3 mm Bulldog Serrefine, Interfocis, Linton) or noxious ipsilateral knee pinch (0.75 cm clamp was placed around the knee). This trial was repeated and pre-conditioned and DNIC responses were calculated as the mean from the two trials. A DNIC response was quantified as an inhibition on mechanically evoked neuronal firing in presence of the conditioning noxious pinch. A 1-min nonstimulation recovery period was allowed between each test, while a 10-min nonstimulation recovery period was allowed between each trial to ensure neuronal responses had returned to baseline (EP n = 19 neurons from 18 animals, EPS n = 13, LP n = 41 neurons from 35 animals, LPS n = 25).

### Knee histology

Following electrophysiology, animals were terminally anaesthetised and knee joints were dissected and fixed in 4% paraformaldehyde (PFA) for 24–48 h. The knees were decalcified in Immunocal^®^ (Quarttet) for approximately 1 week. The knee tissue was then processed with a series of ethanol and wax washes, cut into 10 μM serial sections using a microtome (Microm HM 360) and stained with toluidine blue. Scoring was blinded and carried out by two individuals using a system previously described (Glasson *et al.*, 2010) (Table I). For the maximum score the highest score throughout the entire knee was taken, while the average maximum score is an average of the highest scoring condyle from each slide (EP n = 6, EPS n = 6, LP n = 9, LPS n = 5).

**Table I:** Knee histology scores

Grade	Osteoarthritic damage
0	No damage
0.5	Some loss of toluidine blue staining
1	Small superficial lesions of the articular cartilage
2	Small lesions immediately below the superficial layer of articular cartilage
3	Large lesions or erosion of the articular cartilage covering up to 20% of the condyle
4	Loss of articular cartilage tissue from 20% to 50% of the condyle surface
5	Loss of articular cartilage tissue from 50% to 80% of the condyle surface
6	Loss of articular cartilage tissue from more than 80% of the condyle surface

### Fast blue neuronal tracer

Animals were anaesthetized with ketamine (n = 3) [0.6 ml sterile saline, 0.1 ml medetomidine hydrochloride (1 mg/ml), 0.24 ml ketamine (0.3 mg/ml)], with 250 μL injected interperitoneally per 100 g of rat. 25 μL of Fast Blue (2% w/v in dH_2_O) (Polysciences Europe GmbH) was injected in the knee. The animals were bought back to consciousness with the reversing agent atipamezole (5 mg/ml), where 25 μL was injected subcutaneously per 100 g of animal.

### Immunohistochemistry

Animals were terminally anaesthetised and transcardially perfused with 4% PFA (EP n = 3, EPS n = 3, LP n = 5, LPS n = 4). The ipsilateral L3-L5 dorsal root ganglion (DRG)s were dissected and post-fixed in 4% PFA overnight. Tissue was placed in 30% sucrose overnight, mounted in OCT, frozen in liquid nitrogen and stored at −80°C. Transverse 10 μM sections were cut on a cryostat and placed on SuperFrost Plus slides. Slides were incubated with blocking buffer (10% donkey serum and 0.02% Triton-X-100 in PBS), for 6 h at room temperature. Slides were incubated with primary antibodies (βIII-tubulin - Invitrogen, mouse monoclonal, 1:1000, ATF-3 - Santa Cruz, rabbit polyclonal, 1:500) overnight at room temperature and incubated with secondary antibodies conjugated with Alexa-488 (anti-mouse, 1:1000) (Invitrogen), or Alexa-564 (anti-rabbit, 1:1000) (Invitrogen) for 2 h at room temperature. Slides were mounted with Fluoromount-G and 4 DRG slices per animal were imaged using a Zeiss Imager Z.1 microscope, and neuronal diameter was measured using ImageJ.

### qPCR

Animals were terminally anaesthetised with an overdose of isoflourane and the ipsilateral lumbar dorsal horn and L3-L5 DRGs were dissected (EP n = 4, EPS n = 4, LP n = 4, LPS n = 4), snap frozen in liquid nitrogen and stored at −80°C. RNA was extracted from homogenized tissue using a RNAse microkit (Qiagen). First strand cDNA synthesis was performed on 500 ng RNA using a Superscript III Reverse Transcriptase kit (Invitrogen) according to manufacturers instructions with deoxynucleotide-triphosphates (Promega), and random primers (Promega). mRNA levels of IL-1β, TNFα, IL-6, ATF-3, Iba1, and glial fibrillary acid protein (GFAP) were measured with quantitative PCR using specific primers (Table II) and LightCycler^®^ 480 SYBR Green I master mix (Roche). The mRNA levels were normalized to GAPDH and expressed as either 2-ΛCT values or relative to sham groups.

**Table II:** Primer sequences

Gene	Forward sequence (5‘-3’)	Reverse sequence (5‘-3’)	Source
IL-1β	AGGAGAGACAAGCAACGACA	TTTGGGATCCACACTCTCCAG	Invitrogen
Iba1	TCCCCACCTAAGGCCACCAGC	CGTCTCCTCGGACCACTGGA	Sigma
ATF-3	GGTCGCACTGACTTCTGAGG	CTCTGGCCGCTCTCTGGA	Sigma
TNFα	CGTCGTAGCAAACCACCAAGC	ATGGCGGAGAGGAGGCTGACT	Sigma
IL-6	TCTCTCCGCAAGAGACTTCC	CCGGACTTGTGAAGTAGGGA	Sigma
GFAP	CAACCTCCAGATCCGAGAA	TCTTGAGGTGGCCTTCTGAC	Sigma
GAPDH	CTGCACCACCAACTGCTTAG	TGATGGCATGGACTGTGG	Sigma

### Statistical analysis

Statistical analyses were performed using SPSS v22 (IBM, Armonk). All data plotted represents the mean ± SEM. For behaviour and histological studies when comparing independent samples, a Kruskall–Wallis test was used to compare data from MIA and sham animals. A relationship between behaviour and histology was tested with linear regression analysis, were R^2^ represents the strength of a linear relationship and significance was calculated from an F test. For electrophysiology, statistical differences in neuronal responses with noxious conditioning ear or knee pinch were determined using a two-way repeated-measures ANOVA with Bonferroni post hoc test. For Fast Blue experiments, differences between Fast blue positive neurons in the DRGs was assessed with a Friedmans test. For qPCR experiments, the data expressed relative to sham was tested with a Kruskall–Wallis test and the data expressed as 2-ΛCT values was compared to sham controls with an independent samples *t*-test. Asterisks denote statistically significantly differences (*P < 0.05, **P < 0.01, ***P < 0.001).

## Results

### Pain behaviour and cartilage degradation in MIA animals

**Fig. 1:**
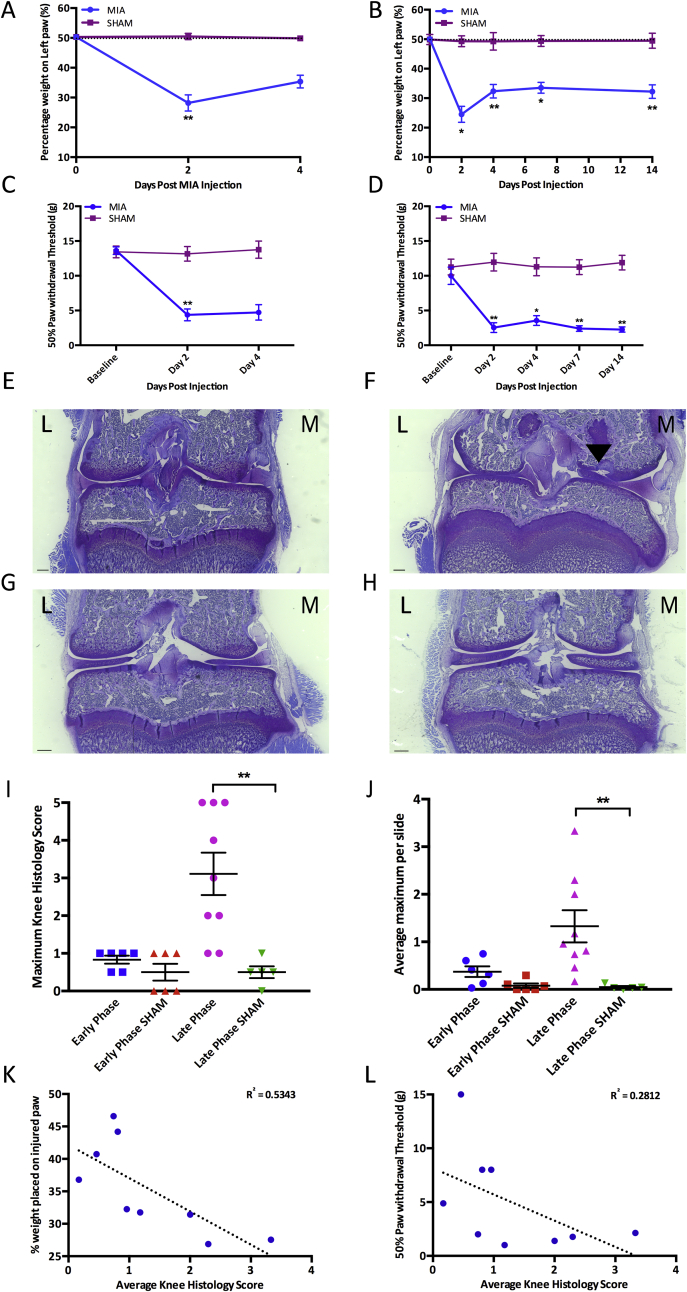
Shows the pain behaviour and knee histology in early phase (EP) and late phase (LP) MIA animals. Scale bars = 500 μM. A) early inflammatory phase (EP) MIA animals place less weight on the injured limb than sham controls (EP n = 15, early phase sham (EPS) n = 12). B) late chronic phases (LP) MIA animals place significantly less weight on the injured limb than sham controls at all time points tested (LP n = 14, late phase sham (LPS) n = 14). C) EP MIA animals have a reduced paw threshold compared to sham controls (EP n = 10, EPS n = 10). D) LP MIA animals have a significantly reduced paw withdrawal threshold than sham controls at all time points tested (LP n = 10, LPS n = 13). E) EP MIA animals display very little cartilage degradation. F) LP MIA animals display extensive cartilage damage. G-H) EP and LP sham animals display no cartilage degradation H) For maximum knee histology score, LP MIA animals have a significantly higher knee histology score than their respective sham control group, while EP MIA animals have similar knee histology scores to their respective sham control group (EP n = 6, EPS n = 6, LP n = 9, LPS n = 5). I) For average knee histology score LP MIA animals have a significantly higher knee histology score than their respective sham control group, but EP MIA animals do not have a significantly higher average knee score than sham controls (EP n = 6, EPS n = 6, LP n = 9, LPS n = 5). J) There is a significant negative linear relationship between average knee histology score and the weight placed on the injured limb in LP MIA animals (n = 9). There is a negative linear relationship between knee histology score and paw withdrawal threshold or weight placed on the injured limb in LP animals (n = 9). (A–H: Kruskall–Wallis. K–L: Linear regression analysis, R^2^ = strength of relationship and *P* value calculated from F-test of overall significance. *P < 0.05, **P < 0.01). EP = EP MIA, EPS = EP sham, LP = LP MIA, LPS = LP sham.

Both EP and LP MIA animals developed mechanical hypersensitivity on the ipsilateral hind paw and avoided putting weight on the injured limb. EP MIA-injected animals demonstrated a reduction of weight placed on the MIA injected limb compared to sham controls [Fig. 1(A)] (Kruskall–Wallis day 2: *P* = 0.001, day 4: *P* = 0.126). LP MIA animals also demonstrated a significant reduction in the weight placed on the injected limb compared to sham controls at all days tested (Kruskall–Wallis day 2: *P* = 0.017, day 4: *P* = 0.001, day 7: *P* = 0.002, day 14: *P* = 0.001) [Fig. 1(B)]. Both EP and LP MIA animals demonstrated a reduced paw withdrawal threshold on the ipsilateral hind paw compared to sham controls (Kruskall–Wallis. EP day 2: *P* = 0.003, day 4: *P* = 0.058, LP day 2: *P* = 0.06, day 4: *P* = 0.025, day 7: *P* = 0.002, day 14: *P* = 0.001), indicating the development of secondary mechanical hyperalgesia [Fig. 1(C) and (D)].

EP MIA animals displayed very little cartilage damage; there was no significant difference in knee scores compared to sham controls (Kruskall–Wallis, max score: *P* = 0.304, average score: *P* = 0.533) [Fig. 1(E), I and (J)]. Histology in LP MIA animals showed extensive cartilage damage [Fig. 1(F)], and knee scores were significantly higher than sham controls (Kruskall–Wallis, max score: *P* = 0.001, average score: *P* = 0.006) [Fig. 1I and (J)].

The MIA model has faced criticism that while it represents the symptoms associated with the development of OA it may not accurately model the joint structural changes; the pain phenotype may be attributed to the neurotoxicity of MIA destroying other tissues surrounding the joint.^3^, ^25^, ^28^ The results from EP MIA animals agree with this concept as rats develop significant pain-like behaviour despite the absence of joint pathology. However, we demonstrate a relationship between cartilage damage and the pain-like behaviour by day 14. Interestingly, in LP MIA animals a negative linear relationship exists between knee scores and weight bearing or paw withdrawal threshold, such that animals with higher knee histology scores place less weight and withdraw at lower mechanical forces on the ipsilateral hind paw (Linear regression analysis, weight bearing: R^2^ = 0.5343, *P* = 0.0253, PWT: R^2^ = 0.2812, *P* = 0.1420) (Fig. 1(K) and L).

### DNIC in MIA animals

**Fig. 2:**
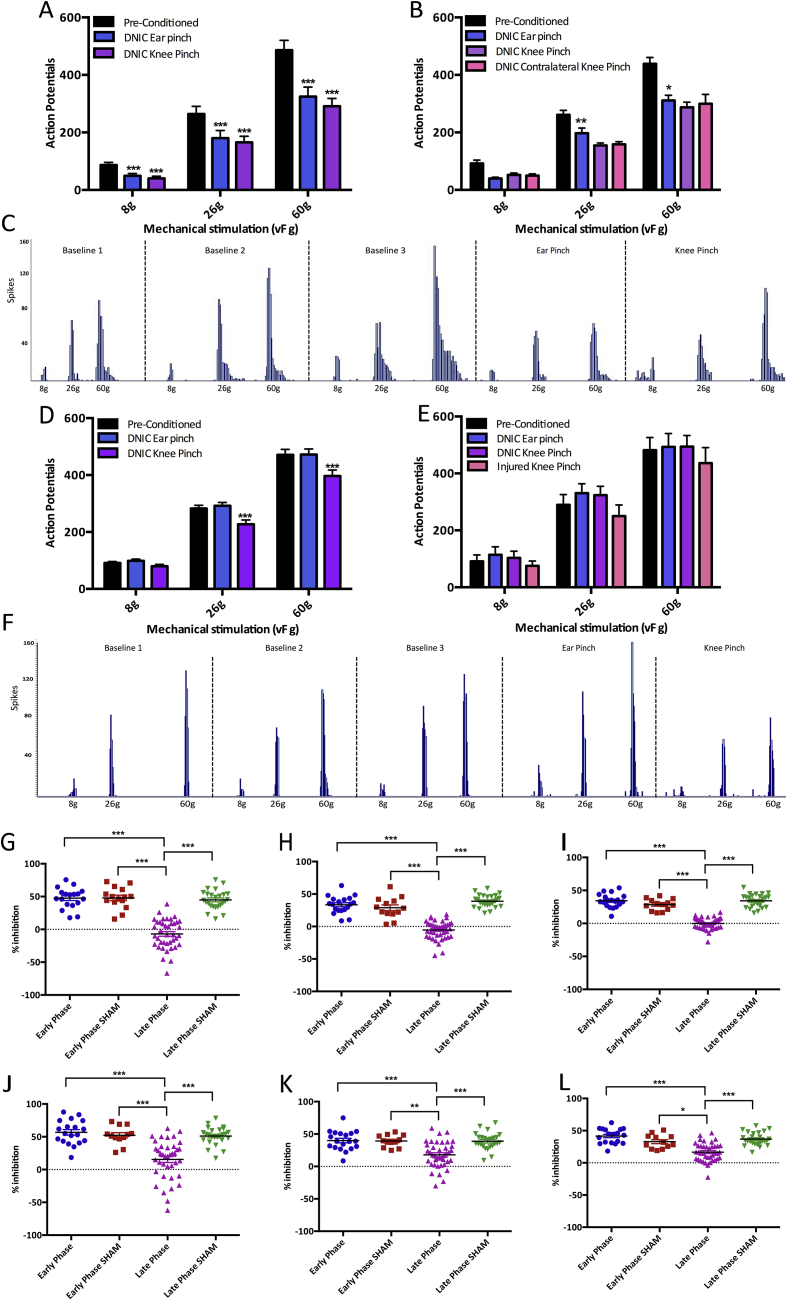
Shows the Diffuse Noxious Inhibitory Controls (DNIC) expression in EP and LP MIA animals. A) A conditioning noxious ipsilateral ear and knee pinch produced a significant reduction in mechanically evoked neuronal firing in ipsilateral wide dynamic range (WDR) neurons in EP MIA animals (n = 19 neurons from 18 animals). B) A conditioning ear pinch and a conditioning pinch placed on both the MIA injured and uninjured knee reduced mechanically evoked neuronal firing in contralateral WDR neurons (n = 5). C) A representative trace from an ipsilateral WDR neuron in an EP MIA animal, showing three baseline responses and a DNIC induced reduction in neuronal firing. D) In LP MIA animals a conditioning noxious ear pinch no longer produces a reduction in neuronal firing in ipsilateral WDR neurons, but there is a significant decrease in neuronal firing with a conditioning noxious knee pinch on the MIA injured knee (n = 41 neurons from 35 animals). E) Electrophysiological recordings from contralateral WDR neurons in LP MIA animals show that a conditioning noxious ear pinch or knee pinch placed on the uninjured knee no longer produce a reduction in neuronal firing, yet a contralateral knee pinch on the MIA injured knee does produce a reduction in neuronal firing (n = 7). F) A representative trace from an ipsilateral WDR neuron in a LP MIA animal, showing three baseline responses, a conditioning noxious ear pinch no longer produces a reduction in neuronal firing yet a conditioning noxious knee pinch does. G-I) The conditioned response as a percentage of the baseline in EP and LP MIA animals and sham controls with a conditioning noxious ear pinch (G = 8 g, H = 26 g, I = 60 g). This shows a consistent reduction in neuronal firing in EP MIA and both sham groups, but no reduction in neuronal firing in LP MIA animals with noxious ear pinch. J-L) The conditioned response as a percentage of the baseline in EP and LP MIA animals and sham controls with a conditioning noxious knee pinch (J = 8 g, K = 26 g, L = 60 g). This shows a consistent reduction in neuronal firing with a conditioning noxious knee pinch in EP and sham groups, while the response is more varied in LP MIA animals with some animals expressing DNIC and some showing no reduction in neuronal firing (EP n = 19 neurons from 18 animals, EPS n = 13, LP n = 41 neurons from 35 animals, LPS n = 25). (A–E: Two-way ANOVA with Bonferroni correction, G–L: Kruskall–Wallis. *P < 0.05, **P < 0.01, ***P < 0.001).

The presence of DNIC was confirmed by a reduction in mechanically evoked neuronal firing, induced by a conditioning noxious ear or knee pinch. In EP animals the conditioning ear and knee pinch considerably reduced neuronal firing for all mechanical stimulations in both ipsilateral and contralateral WDR neurons (Two way ANOVA, Ipsi: 8 g: ear: *P* < 0.0001, knee: *P* < 0.0001, 26 g: ear: *P* < 0.001, knee: *P* < 0.0001, 60 g: ear: *P* < 0.0001, knee: *P* < 0.0001, Contra: 8 g ear: *P* = 0.072, uninjured knee: *P* = 0.802, MIA knee: *P* = 0.793, 26 g: ear: *P* = 0.004, uninjured knee: *P* = 0.171, MIA knee: *P* = 0.214, 60 g: ear *P* = 0.038, uninjured knee: *P* = 0.196, MIA knee: *P* = 0.498) [Fig. 2(A), (B) and (C)]. Yet, in LP MIA animals there was consistently no reduction in mechanically evoked neuronal firing with a conditioning noxious ear pinch, in either ipsilateral or contralateral WDR neurons, indicating an alteration in the endogenous inhibitory system (Two way ANOVA, Ipsi: 8 g: *P* = 0.184, 26 g: *P* = 0.456, 60 g: *P* = 1, Contra: 8 g: *P* = 0.683, 26 g: *P* = 0.084, 60 g: *P* = 1) (Fig. 2(D), (E), (F), (G), (H) and I). Remarkably, a reduction in neuronal firing could be induced in some LP animals with a conditioning noxious knee pinch, indicating the DNIC system is not completely abolished if the conditioning stimulus is high (Two way ANOVA, Ipsi: 8 g: *P* = 0.095, 26 g: *P* < 0.0001, 60 g: *P* < 0.0001, Contra: 8 g: *P* = 0.866, 26 g: *P* = 0.0476, 60 g: *P* = 0.346) (Fig. 2(D), (E), (F), (J), (K) and L).

### Fast Blue neuronal tracer

**Fig. 3:**
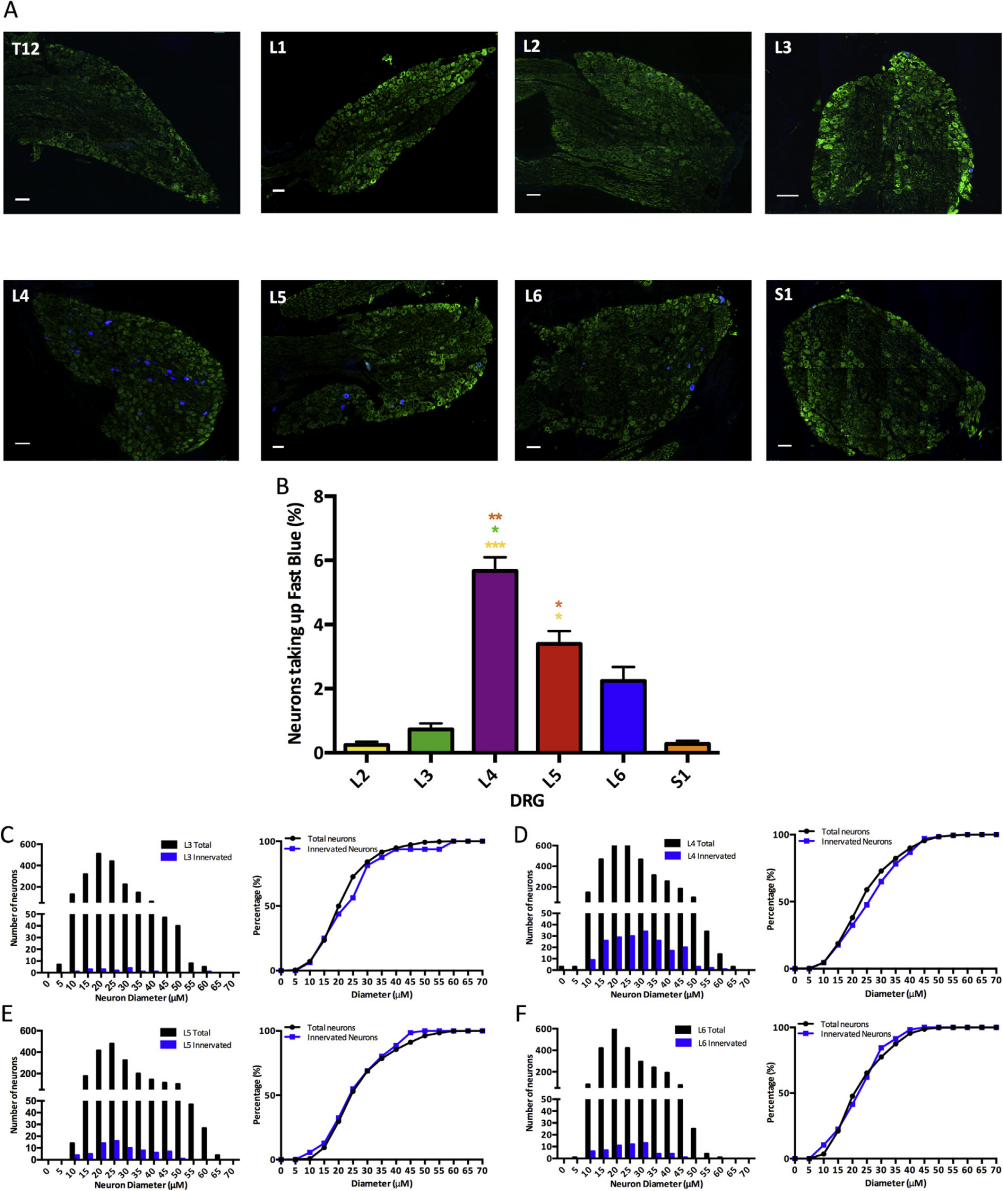
Shows neurons in ipsilateral lumbar DRGs taking up a Fast blue neuronal tracer. Green = βIII-tubulin, Blue = Fast blue neuronal tracer, all scale bars = 100 μM. A) T10-S1 DRGs B) There are a significantly higher number of Fast blue positive neurons in L4 and L5 DRGs (n = 3). C) The size distribution of neurons taking up the neuronal tracer is similar to the overall size distribution, indicating both small and large neurons innervate the knee (B: Friedmans. *P < 0.05, **P < 0.01, ***P < 0.001).

Peripheral afferent fibres that took up Fast Blue projected from L2 to S1 DRGs [Fig. 3(C)]. The L4 and L5 DRGs had significantly higher percentages of neurons showing positive staining for Fast Blue, with L4 DRGs having 5.7% of neurons stained (Friedmans, vL2: *P* < 0.0001, vL3: *P* = 0.025, vS1: *P* = 0.001) and L5 DRGs having 3.4% of neurons stained (Friedmans, vL2: *P* = 0.013, vS1: *P* = 0.40). Cumulative sum analysis indicated that for L3-L6 DRGs the size distribution for Fast Blue positive neurons was very similar to the size distribution of the total neurons within the DRG [Fig. 3(C), (D), (E) and (F)], indicating that both small and large diameter peripheral afferent fibres innervate the knee. The contralateral DRGs did not show positive Fast blue staining (data not shown).

### ATF3 expression in MIA animals

**Fig. 4:**
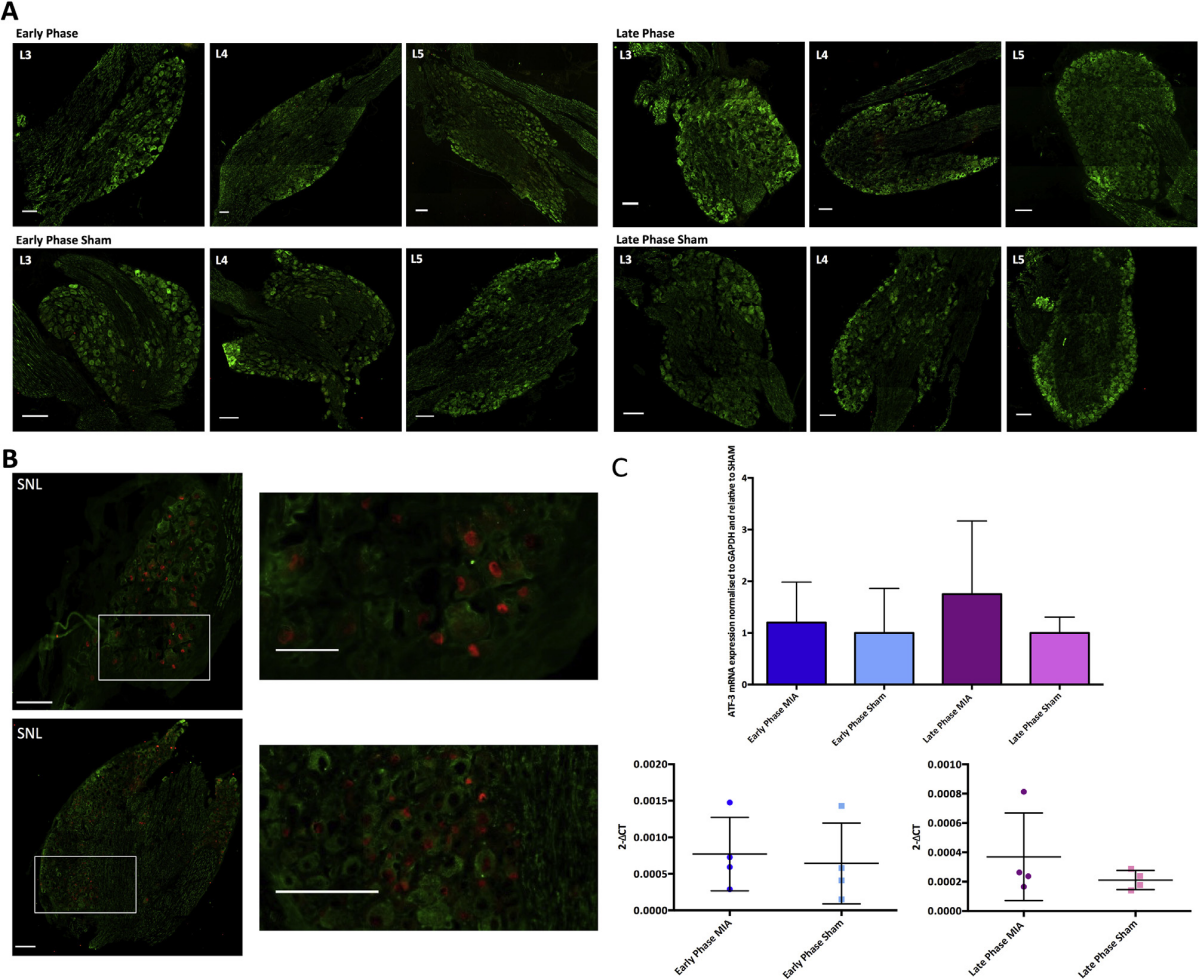
Shows ATF-3 expression in EP and LP MIA animals. Green = βIII-tubulin, Red = ATF-3, all scale bars = 100 μM. A) There was no positive ATF-3 staining in ipsilateral lumbar DRGs in either MIA or sham group (EP n = 3, EPS n = 3, LP n = 5, LPS n = 4). B) There was extensive positive ATF-3 staining in ipsilateral lumbar DRGs taken from animals with neuropathy (n = 2). C) The levels of ATF-3 mRNA expression in ipsilateral lumbar DRGs are similar between MIA animals and sham controls (EP n = 4, EPS n = 3, LP n = 4, LPS n = 4) (C: mRNA expression normalized to sham controls was tested with a Kruskall–Wallis, for mRNA expression expressed as 2-ΔCT values an independent samples *t*-test was used).

ATF-3 is a transcription factor, which is upregulated in response to stressful stimuli and is considered a sensitive marker for detecting neuronal damage. 28, 29 No ATF-3 staining was observed in L3-L5 DRGs taken from either EP (day 4) or LP (day 14) MIA animals [Fig. 4(A)].^25^ Furthermore, there was no significant increase in the mRNA expression of ATF-3 in the ipsilateral L3-L5 DRGs in EP or LP MIA animals compared to sham controls (Kruskall–Wallis: EP v Sham *P* = 0.094, LP v Sham *P* = 0.197, independent-samples *t*-test: EP: *P* = 0.743, LP: *P* = 0.339) [Fig. 4(C)], suggesting MIA is not causing substantial nerve trauma in MIA animals. Confirming the validity of the antibody, ATF-3 expression was observed in the nucleus of lumbar DRG cells taken from rats with neuropathy [Fig. 4(B)].

### Central inflammatory changes in the dorsal horn

**Fig. 5:**
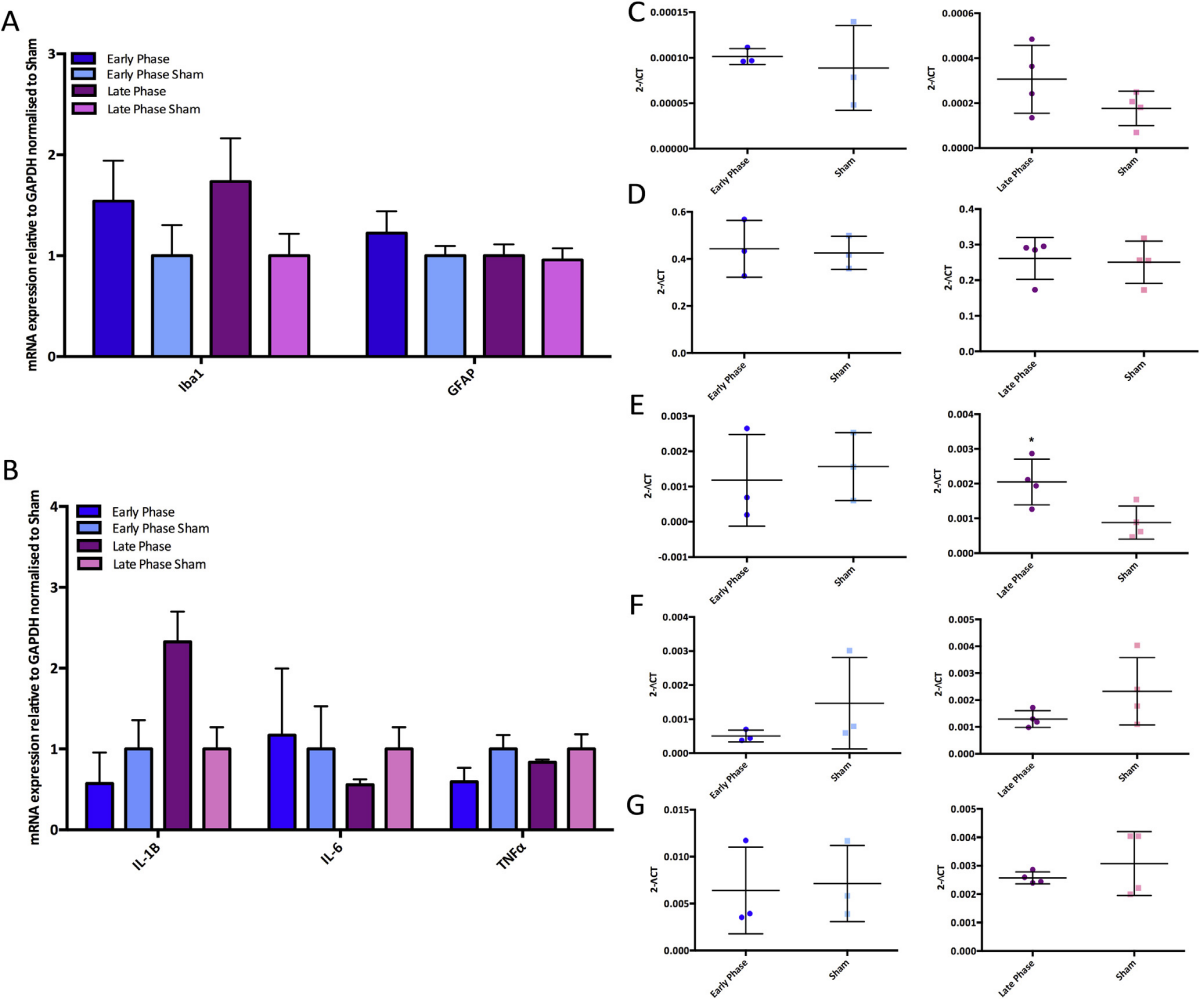
Shows mRNA expression of glia cells and cytokines in the lumbar dorsal horn of EP and LP MIA animals (EP n = 4, EPS n = 4, LP n = 4, LPS n = 4). A) mRNA expression of Iba1 and glial fibrillary acid protein (GFAP) normalized to sham. B) mRNA expression of pro-inflammatory cytokines normalized to sham. C) There is a weak rise in Iba1 mRNA expression in LP MIA animals. D) GFAP mRNA levels remain similar between MIA and sham groups. E) There is a significant increase in IL-1β mRNA expression in LP MIA animals. F) IL-6 mRNA levels remain similar between MIA and sham groups. G) TNFα mRNA levels remain similar between MIA and sham groups (A–B: for mRNA expression normalized to sham controls a Kruskall–Wallis test was used, C–G: for mRNA expression expressed as 2-ΔCT values an independent samples *t*-test was used. *P < 0.05).

An increased number of activated microglia has previously been reported in the ipsilateral SC of MIA animals, which is thought to contribute to the development of central sensitisation.^25^, ^30^ Therefore, the mRNA expression of the microglia marker Ionized calcium binding adaptor molecule 1 (Iba1) and astrocyte marker GFAP were assessed in the dorsal horn using qPCR in MIA and sham animals. There was weak evidence for a rise in Iba1 mRNA expression in LP MIA animals but this effect was not significant (EP: *P* = 0.669, LP: *P* = 0.176) [Fig. 5(A) and (C)]. We observed no difference in GFAP mRNA expression levels between MIA and sham animals (EP: *P* = 0.839, LP: *P* = 0.805), indicating there is no increase in the number of astrocytes in the dorsal horn following MIA injection at these time points [Fig. 5(A) and (D)].

The increased release of proinflammatory cytokines from activated spinal glia, in particular IL-1β, TNFα, and IL6 have been demonstrated following peripheral injury.^31^, ^32^ The mRNA expression of IL-6 and TNFα in the dorsal horn remained relatively constant in both MIA and sham groups (IL-6; EP: *P* = 0.285, LP: *P* = 0.161, TNFα; EP: *P* = 0.846, LP: *P* = 0.439) [Fig. 5(B), (F) and (G)]. However, there was a significant increase in the mRNA expression of IL-1β in the ipsilateral dorsal horn of LP MIA animals compared to sham (*P* = 0.028), but this was not found in EP MIA animals (*P* = 0.699).

## Discussion

We demonstrate that EP MIA animals develop significant pain-like behaviour despite histology indicating a lack of cartilage degradation, which agrees with previous reports.^33^, ^34^ MIA causes an initial inflammatory infiltrate of monocytes, neutrophils and basophils in the joint, peaking around day 3, thus pain behaviour in EP animals may be due to the general toxicity of MIA causing an inflammatory response in the joint.^6^, ^10^ However, only cartilage damage was scored with histology and since cartilage is both anueral and avascular it cannot be the direct cause of pain.^35^ Therefore, the pain behaviour observed in EP MIA animals may be a result of damage to other joint structures, such as the synovium. The pain-like behaviour observed in the EP MIA model may not be a result of joint pathology that is typical of human OA, and representative of the mechanisms driving OA associated pain. LP MIA animals present with structural joint damage, including loss of articular cartilage and in some cases exposure of subchondral bone, which has been demonstrated to be associated with the resultant pain behaviour.^36^ Indeed, a linear relationship was shown between knee histology score and the weight placed on the ipsilateral limb. Although this is insufficient evidence to demonstrate causality, this does agree with previous evidence that MIA-induced joint pathology depends upon time, with later time points more accurately modeling the structural joint pathology and pain that occur in human OA.^10^, ^31^

We show that EP MIA animals have a normally functioning DNIC as both concurrent noxious ear and knee pinch produced a significant reduction in mechanically evoked neuronal firing. On the other hand, there was a loss of DNIC in both ipsilateral contralateral WDR neurons of LP MIA animals as the conditioning ear pinch no longer produced a reduction in neuronal firing, indicating a bilateral loss of inhibitory control, which is similar to that observed in SNL animals.^13^ A functional DNIC system relies on a balance between descending noradrenergic inhibitory pathways and facilitatory serotonergic pathways,^13^, ^19^ so we suggest an imbalance in descending controls develops in LP MIA animals.^24^, ^37^ Specifically, a reduction in descending noradrenergic inhibitory controls and an enhanced descending serotonergic facilitatory drive acting at 5-HT_3_ receptors in the SC has been demonstrated in LP 2 mg MIA animals.^24^, ^37^ Furthermore, while NSAIDs are effective at relieving hyperalgesia and allodynia in EP MIA animals, they are ineffective in LP MIA animals, which further indicates central changes.^7^ Overall, the pain behaviour in EP animals is likely a result of peripheral sensitisation, yet in LP animals central changes lead to an imbalance in inhibitory and faciliatory descending controls from the brainstem.

Remarkably, in some LP MIA animals a reduction in neuronal firing was observed when the conditioning noxious pinch was placed on the injured knee. This indicates that the DNIC system is not completely abolished and we propose that the imbalance in descending controls is masking the expression of DNIC, which agrees with previous studies demonstrating DNIC can be re-established pharmacologically.^13^, ^19^ Previous studies using electrophysiological recordings from joint afferents have demonstrated MIA produced a graded sensitisation and increased firing rate, while a Nav1.8 channel blocker reduced the firing rate of joint afferents in response to noxious rotation of the joint.^38^, ^39^ Thus noxious conditioning stimulation of the MIA injured knee produces enhanced firing of joint afferents and an increased transduction of the nocicieptive signal to the dorsal horn. Therefore, the conditioning MIA injured knee pinch may now produce a sufficient nociceptive signal to activate suppressed descending inhibitory controls and produce a small level of neuronal inhibition.

Histology studies indicated that in areas of extreme cartilage degradation there was exposure of the subchondral bone, which may lead to peripheral nerve damage. This study demonstrated that the majority of knee joint afferents were localized in L4 and L5 DRGs, while a previous study found that the majority of subchondral bone afferents are localized in the L3 DRGs.^40^ Interestingly, no upregulated ATF-3 mRNA expression could be identified in ipsilateral L3-L5 DRGs in either EP or LP MIA groups, suggesting a lack of nerve trauma in knee joint or subchondral bone afferents. This contrasts with previous MIA studies that identified substantial ATF-3 expression in lumbar DRGS. However, the time post MIA injection when ATF-3 expression arises differs between studies, with one identifying ATF-3 staining as soon as day 3, and others reporting that ATF-3 upregulation only occurred later in the model.^25^, ^41^, ^42^ Furthermore, no study reported an increase in ATF-3 positive neurons in the L3 DRGs, indicating the reported neuropathic component was not a result of damage to peripheral afferents innervating the subchondral bone.^25^, ^40^, ^41^, ^42^ Furthermore, Thakur *et al.* reported that 20% of L5 DRG cells bodies showed positive staining for ATF-3, whereas the neuronal tracer experiment in this study indicated that only 4–6% of L4 and L5 neurons innervated the knee.^25^ In addition, ATF-3 staining in the ventral horn indicated damage to motor neurons surrounding the knee, suggesting leakage of MIA from the intraarticular space, yet we identified no ATF-3 positive staining in the ventral horn (data not shown).^25^ These differences cannot be attributed to MIA dose or volume injected, but could be down to precision with the injection technique. In agreement with our findings, a mouse study found few ATF-3 positive cell bodies in L3-L5 DRGs 7 days after MIA injection, as only 2.1% of cell bodies displayed positive staining and the authors concluded there was no evidence to suggest MIA induced sensory neuron damage.^9^ Overall, the lack of ATF-3 expression in this study suggests there is no MIA induced neuronal damage and that an alternative mechanism must be driving central changes in LP animals.

Glial cells, a major component of the central nervous system, can respond to peripheral tissue damage, releasing factors that interact with neurons to regulate their function.^43^, ^44^ Previous studies reported an increase in microglia proliferation and activation of in SC following MIA injection^9^, ^25^, ^30^. Importantly, increase in the number of microglial cells was reported to be increased no earlier than 28 days after MIA injection^9^. Indeed, in our study we found no changes in Iba1 mRNA levels in the SC 14d after MIA injection, suggesting no increase in the number of microglia cells up to this time in this model. Beyond proliferation, when activated, microglia can modulate the function of spinal neurons by releasing pro-inflammatory cytokines^34^, ^45^. Here, we show a significant increase in IL-1β mRNA expression in the SC dorsal horn 14d after MIA injection. Notably, it has been suggested that IL-1β can influence synaptic transmission by enhancing AMPA and NMDA as well as suppressing GABA and glycine induced currents in the SC dorsal horn.^46^ It is believed therefore that microgliosis and the subsequent release of IL-1β may be contributing the development of central sensitisation,^46^ a feature we speculate to be occurring in the model used in our study. Further, we report no increase in the mRNA expression levels of the astrocytic marker GFAP in the MIA animals, which is in agreement with previous findings where an increase in GFAP expression can only be observed at later time points of 28d post MIA injection.^30^ Investigating the molecular and cellular changes in the microglia at the single cell and population levels^48^, ^49^ after MIA injection, which are beyond the scope of this study, would be necessary to further characterize microglia proliferation and activation and potentially target the microgliosis observed in this model.

Many OA patients develop referred pain, and a subset are left with persistent pain following total joint replacement surgery, indicating OA associated chronic pain cannot always be explained by purely peripheral mechanisms.^47^, ^50^ An alteration in descending controls, together with central sensitisation may be responsible for this persistent pain. Indeed, clinical studies in OA patients have shown a loss of CPM and an abnormal descending system from the PAG to the SC.^21^, ^50^ This study agrees with pre-existing evidence that an imbalance in inhibitory and facilitatory descending controls develops as the MIA model progresses.^24^, ^37^ Overall, the development of chronic pain as a result of OA may be attributed to both peripheral mechanisms due to joint damage, and subsequent centrally mediated mechanisms. Both mechanisms should be considered when deciding on the appropriate analgesics for patients.

## Contributions

Stevie Lockwood: Collection analysis and interpretation of the data, and drafting of the article.

Douglas Lopes: Conception and design of the study.

Stephen McMahon: Conception and design of the study, obtaining of funding and final approval of the article.

Anthony Dickenson: Conception and design of the study, obtaining of funding and final approval of the article.

## Conflict of interest

The authors have no conflict of interest to declare.

## Role of the funding source

This study was funded by the Wellcome Trust Pain Consortium (102645—Defining pain circuitry in health and disease).
