# Supplementary material for: Characterisation of peripheral and central components of the rat monoiodoacetate model of Osteoarthritis
Source: Osteoarthritis Cartilage. 2019 Apr;27(4):712–22. doi: 10.1016/j.joca.2018.12.017 (PMC6444078; doi:10.1016/j.joca.2018.12.017)
Supplement: Multimedia component 1 [file mmc1.docx]

Table S1. Cohen’s D value for longitudinal behavioural data

| Comparison | Cohen’s D |
| --- | --- |
| **Weight bearing: early phase** | |
| MIA v Sham Baseline | 0.01 |
| MIA v Sham Day 2 | 2.95 |
| MIA v Sham Day 4 | 3.33 |
| **Weight bearing: late phase** | |
| MIA v Sham Baseline | 0.11 |
| MIA v Sham Day 2 | 5.07 |
| MIA v Sham Day 4 | 2.65 |
| MIA v Sham Day 7 | 3.14 |
| MIA v Sham Day 14 | 2.70 |
| **Paw withdrawal threshold: early phase** | |
| MIA v Sham Baseline | 0.08 |
| MIA v Sham Day 2 | 2.90 |
| MIA v Sham Day 4 | 3.30 |
| **Paw withdrawal threshold: late phase** | |
| MIA v Sham Baseline | 0.31 |
| MIA v Sham Day 2 | 2.97 |
| MIA v Sham Day 4 | 3.13 |
| MIA v Sham Day 7 | 3.10 |
| MIA v Sham Day 14 | 3.43 |
